# Using GPS collars to investigate the frequency and behavioural outcomes of intraspecific interactions among carnivores: A case study of male cheetahs in the Maasai Mara, Kenya

**DOI:** 10.1371/journal.pone.0213910

**Published:** 2019-04-03

**Authors:** Femke Broekhuis, Emily K. Madsen, Kosiom Keiwua, David W. Macdonald

**Affiliations:** 1 Kenya Wildlife Trust, Nairobi, Kenya; 2 Wildlife Conservation Research Unit, Department of Zoology, University of Oxford, Recanati-Kaplan Centre, Tubney, United Kingdom; 3 Centre for Biodiversity and Environmental Research, University College London, London, United Kingdom; University of Tasmania, AUSTRALIA

## Abstract

Intraspecific interactions between individuals or groups of individuals of the same species are an important component of population dynamics. Interactions can be static, such as spatial overlap, or dynamic based on the interactions of movements, and can be mediated through communication, such as the deployment of scent marks. Interactions and their behavioural outcomes can be difficult to determine, especially for species that live at low densities. With the use of GPS collars we quantify both static and dynamic interactions between male cheetahs (*Acinonyx jubatus*) and the behavioural outcomes. The 99% home-ranges of males overlapped significantly while there was little overlap of the 50% home-ranges. Despite this overlap, male cheetahs rarely came into close proximity of one another, possibly because presence was communicated through frequent visits to marking posts. The minimum distance between individuals in a dyad ranged from 89m to 196m but the average proximity between individuals ranged from 17,145 ± 6,865m to 26,367 ± 11,288m. Possible interactions took place more frequently at night than by day and occurred mostly in the 50% home-range of one individual of a dyad or where cores of both individuals overlapped. After a possible encounter male cheetahs stayed in close proximity to each other for up to 6 hours, which could be the result of a territory defence strategy or the presence of a receptive female. We believe that one of the encounters between a singleton and a 5-male coalition resulted in the death of the singleton. Our results give new insights into cheetah interactions, which could help our understanding of ecological processes such as disease transmission.

## Introduction

Intraspecific interactions, or interactions between members of the same species, are an important component of population dynamics as they play a role in sociality [1], mating events [2], disease transmission [3, 4] and competition which could influence access to resources [5, 6], spatial organisation [7, 8] and mortality [9]. Interactions, which can be defined as “actions directed towards, or affecting, the behaviour of another animal” (sensu [10]), can be categorised into two groups; static or indirect interactions and dynamic or direct interactions [11]. Static interactions lack a temporal element and do not take into account the proximity between individuals. For example, individuals could use similar areas, but at different times. Static interactions can be determined by quantifying the amount of spatial overlap, which can give an indication as to the possibility of dynamic interactions occurring [12]. Unlike static interactions, dynamic interactions (also referred to as encounters or associations) include a temporal component and are based on the spatial proximity of simultaneous locations of individuals. The nature of dynamic interactions can differ and can include mating events or the sharing or defending of resources [2, 13]. Interactions can be mediated through communication, for example olfaction or vocalisation, as this can attract mates or allow conspecifics to assess potential threats which could minimise the occurrence of costly, potentially fatal, encounters [14–16]. When interactions occur they can elicit a change in movement and spacing behaviour, which can vary depending on the nature of the interaction [17, 18].

The likelihood, frequency and outcomes of interactions can be influenced by social structure which can differ significantly amongst species [19]. Felids, for example, are often considered to be predominantly solitary [20] yet the sociality of felids lies on a continuum with lions (*Panthera leo*), which live in social groups, on one end of this spectrum [21]. Even felid species that are often believed to be solitary can engage in social interactions (e.g. [13]). In some species these associations occur occasionally, such as at kills sites [13], whereas in other species these can be more enduring. In cheetahs (*Acinonyx jubatus*), for example, females are solitary, unless they are accompanied by dependent cubs, but male cheetahs can either be solitary or form stable, same-sex groups known as coalitions [22]. Coalitions generally consist of two to three related or unrelated individuals, but a rare five-male coalition has been seen in the Maasai Mara, Kenya (this study). The land tenure system of male cheetahs can broadly be categorised into two groups: floaters, who roam over vast areas that they do not defend, and resident males, who defend small territories possibly based on access to resources such as females [22, 23]. Territorial boundaries can however be fluid [24] and it is believed that cheetahs use a ‘time-share’ approach [25] where territories and home-ranges can overlap but where direct interactions between cheetahs are minimised through olfactory communication. Territorial males advertise their presence by scent marking (urinating and defecating) on marking posts which are usually prominent landscape features such as termite mounds, logs or trees [23, 26]. Despite cheetahs communicating their presence, males can encounter one another and encounters can range from passive [25] to acutely aggressive [27].

Until recently, research on the spatial organisation of male cheetahs has mostly been based on VHF telemetry or behavioural observations (e.g. [22, 27, 28]). These methods of data collection, while informative, make it difficult to continuously monitor several individuals at a time. In addition, while encounters between males have been observed, it is unknown how often they occur and what the behavioural outcomes are [25, 27]. This paucity of data is partly because cheetahs live at low densities and interactions are therefore difficult to observe. However, with the help of data loggers, such as GPS collars, it is possible to detect and quantify interactions when multiple individuals are tagged simultaneously (e.g. [17]).

Here we investigate interactions between male cheetahs using location data collected with GPS collars by investigating 1) static interactions by quantifying spatial overlap and visits to marking posts to determine the frequency of indirect interactions to try and understand the role that marking posts play in cheetah ecology, 2) dynamic interactions by quantifying the proximity between different individuals and 3) the outcomes of possible interactions in terms of movement behaviour and mortalities. Based on previous research we predict that males will overlap spatially but that there will be little overlap of the core areas [29]. We also predict that marking posts are frequently visited by both individuals in a dyad, i.e. pair of cheetahs, and that occasions where individuals of the dyad are in close proximity to each other are infrequent. Because encounters between males can be aggressive [27] we predict that the movement behaviour after a possible encounter would indicate avoidance behaviour (moving away from the encounter location, moving away from one another, increased distance travelled and decreased path tortuosity).

## Methods

### Study area

The study was conducted in the Maasai Mara, in the southwest of Kenya (centred at 1°S and 35°E), which is part of the larger Serengeti-Mara ecosystem. The study area (~2,600 km^2^) included the Maasai Mara National Reserve and the surrounding wildlife conservancies. The area experiences one wet season spanning from November to June and one dry season spanning from July to October [30]. After the wet season, the long grass attracts large numbers of migratory ungulates, including the white-bearded wildebeest (*Connochaetes taurinus*) and the common zebra (*Equus quagga*), from the Serengeti in Tanzania. Throughout the year there is an abundance of cheetah prey including resident white-bearded wildebeest, Thomson’s gazelle (*Eudorcas thomsonii*), Grant’s gazelle (*Nanger granti*) and impala (*Aepyceros melampus*) [31]. The habitat in the Maasai Mara varies, ranging from open grasslands and shrubland, to riverine forests found along the major rivers and their tributaries [32]. The open grassland plains, which are dominated by red oat grass (*Themeda triandra*), are mostly found toward the south and west of the study area, while the north and north-east consist mostly of Croton thickets (*Croton dichogamous*) and Vachellia woodlands (*Vachellia drepanolobium* and *V*. *gerrardii*).

### Cheetah collaring

Global Positioning System (GPS) satellite collars (African Wildlife Tracking - www.awt.co.za) were fitted on four adult male cheetahs between 19^th^ October 2016 and 9^th^ February 2018. In compliance with Kenyan law, all immobilizations for deployment/removal of collars were performed by a Kenya Wildlife Service veterinarian. Cheetahs were free-darted and immobilized using a combination of ketamine (2–2.5 mg/kg) and medetomidine (0.07 mg/kg), remotely administered by a Dan-Inject CO_2_ rifle (Dan-Inject, Denmark), and reversed with atipamezole (0.3 mg/ml; following [33]). Sedation time was kept to a minimum, typically less than 1 hour. After immobilization all cheetahs recovered fully, showing no signs of distress and no apparent side effects were observed in the short- and long-term. Collars, which were only fitted on adults, weighed 400 grams which is the recommended weight for cheetah collars [34]. All collars were removed if they malfunctioned or if the batteries were low. The animal handling protocols used conformed to the standards of the American Society of Mammalogists [35] and permissions to deploy collars were provided by the Kenya Wildlife Service (Permit No.: KWS/BRM/5001) and the National Commission for Science, Technology and Innovation (Permit No.: NACOSTI/P/16/69633/10821).

The collared males were all singletons, except for one male (M03), who was part of a five-male coalition. Over an 18 month period, the five-male coalition were sighted on 73 occasions and only on one occasion did the coalition separate for a period of <24 hours. We therefore collected data on a total of eight individuals in four social groups. While this is a relatively small sample size, it is a quarter of the entire population as there are approximately 32 adults within the study area [36]. The collars collected GPS coordinates every three hours (00h, 03h, 06h, 09h, 12h, 15h, 18h and 21h) and when there was satellite communication, data were uploaded on a daily basis at 06h. On average, the collars were deployed for 285 days ranging from 115 to 349 days (Table 1). For each pair of cheetahs (hereafter referred to as a dyad), we only used simultaneously collected data for the analyses.

**Table 1:** Overview of the male cheetahs that were collared. Dates are presented in dd/mm/yyyy.

Cheetah ID	Group composition	Status˄	Begin date	End date	Total no. of days
M01	Singleton	Territorial	21/10/2016	01/10/2017	345
M02	Singleton	Floater	25/02/2017	09/02/2018	349
M03	5-male coalition	Transitioning	11/03/2017	03/02/2018	329
M04	Singleton	Territorial	19/10/2016	11/02/2017	115

˄ Definition based on findings by [23].

### Data processing and analysis

#### Static interactions

To determine the static interactions between male cheetahs we calculated their space use and the amount of overlap for each dyad to determine the possibility that individuals could encounter each other either directly or indirectly. Space use for each individual per dyad was based on their utilisation distributions, which is the distribution of an individual’s locations over time [37], using the *adehabitat* package [38] in R [39]. To calculate the utilisation distributions we used a fixed kernel density estimate using a bivariate normal kernel. We used the reference bandwidth parameter (h_ref_) as the smoothing factor unless h_ref_ > 1000 then we used 80% of the h_ref_ to minimise over-smoothing of the data. Using the resulting utilisation distribution, we determined both the 99% and 50% kernels which respectively represent an individual’s total space use and their core areas. For each dyad we then calculated the amount of overlap of the 99% kernels and the 50% kernels.

Marking posts are used by male cheetahs to communicate their presence to conspecifics. Using the methods described by [29], we located marking posts based on a cluster analysis using data from the GPS collars and opportunistically when conducting fieldwork. For each dyad, we determined how many marking posts were found within the 99% kernel overlap. We used the *recurse* package [40] to calculate 1) how many marking posts were visited by each individual and 2) how many marking posts were visited by both individuals in a dyad (hereafter referred to as mutual marking posts). We classified a visit when a cheetah came within 500m (half the average step-length, see Dynamic interaction for details) of a marking post. For the mutual marking posts we calculated 1) the time between individual visits and 2) the time spent within 500m of a mutual marking post by each individual and tested whether there were differences between individuals. In addition, we calculated the time between visits from two different individuals i.e. we calculated how long it took for individual *x* to visit a mutual marking post once individual *y* had visited and vice versa. We then tested whether the time it took for an individual to visit a marking post once the other individual had been there differed between the individuals. We tested the data for normality using the Shapiro–Wilk test and used a t-test if the data were normally distributed and a Wilcoxon test if they were not. If the data were not normally distributed then we provided the median ± median absolute deviation in addition to the mean ± standard deviation.

#### Dynamic interaction

We first explored the movement of the different individuals using the *moveVis* package [41]. Then, to determine whether interactions between two individuals in a dyad were likely to occur, we calculated the proximity between simultaneous locations using the *wildlifeDI* package [42]. The 3hr resolution of the data is quite coarse so we used different proximity thresholds to group possible encounters based on the average 3-hour step-length which was 1,021m ± 1,487m (mean ± standard deviation). We used four proximity thresholds: <500m, <1000m, <1500m and <2000m which correspond to 0.5, 1, 1.5 and 2 times the average step-length. We then determined whether these possible encounters took place at night (fixes at 21hr, 00hr, 03hr or 06hr) or during the day (fixes at 09hr, 12hr, 15hr or 18hr), whether they occurred within 50% kernels and the distance to the nearest known marking post. The half-way points between the two individuals were used as the estimated location where a possible encounter occurred.

#### Encounter outcomes

The GPS data were examined once every few days. If any unusual behaviour was detected, such as no movement after a possible encounter, the field team would investigate to establish whether any injuries or deaths occurred as a result. In addition, for each possible encounter we compared the behaviour before to the behaviour after at four different time lags; 3hrs, 6hrs, 12hrs and 24hrs. First, we calculated the distance between two individuals before and after a possible encounter to determine whether males moved away from one another after an encounter. Then, per dyad, we calculated 1) the distance to the encounter location for each individual, 2) the distance travelled per individual and 3) the path tortuosity, or straightness. Path tortuosity was calculated by dividing the net displacement by the total distance travelled. A value of around 1 would indicate that the individual travelled in a straight line whereas a value <1 would be indicative of a tortuous path. We compared the proximity of the two individuals within a dyad, distance to encounter location, distance travelled and tortuosity before and after a possible encounter. We tested the data for normality using the Shapiro–Wilk test and used a t-test if the data were normally distributed and a Wilcoxon test if they were not.

## Results

In total, we secured simultaneous data on four male cheetah dyads ranging from 113 to 329 days per dyad. One individual (M01) was part of three of the four dyads, two individuals (M02 and M03) were part of two dyads and one individual (M04) was part of one dyad (Table 2).

**Table 2:** Summary of static interactions between male cheetah dyads in the Maasai Mara, Kenya. For each dyad, i.e. pair of individuals, only simultaneous data were used.

		Dyad 1		Dyad 2		Dyad 3		Dyad 4	
Individuals		M01	M02	M01	M03	M02	M03	M01	M04
Number of days of simultaneous data		218		204		329		113	
Missed fixes (%)		34 (2.00%)	32 (1.88%)	34 (2.14%)	65 (4.18%)	56 (2.20%)	99 (3.95)	21 (2.38%)	28 (3.27%)
99% kernel	Area	253 km^2^	1903 km^2^	260 km^2^	1384 km^2^	1838 km^2^	1342 km^2^	296 km^2^	347 km^2^
	Overlap area	185 km^2^		260 km^2^		997 km^2^		24 km^2^	
	% overlap	73%	10%	100%	19%	54%	74%	15%	34%
50% kernel	Area	11 km^2^	359 km^2^	12 km^2^	236 km^2^	238 km^2^	139 km^2^	19 km^2^	42 km^2^
	Overlap area	0.42 km^2^		0.60 km^2^		67 km^2^		0 km^2^	
	% overlap	3.79%	0.12%	5.07%	0.25%	28%	48%	0%	0%

### Static interaction

**Fig 1:**
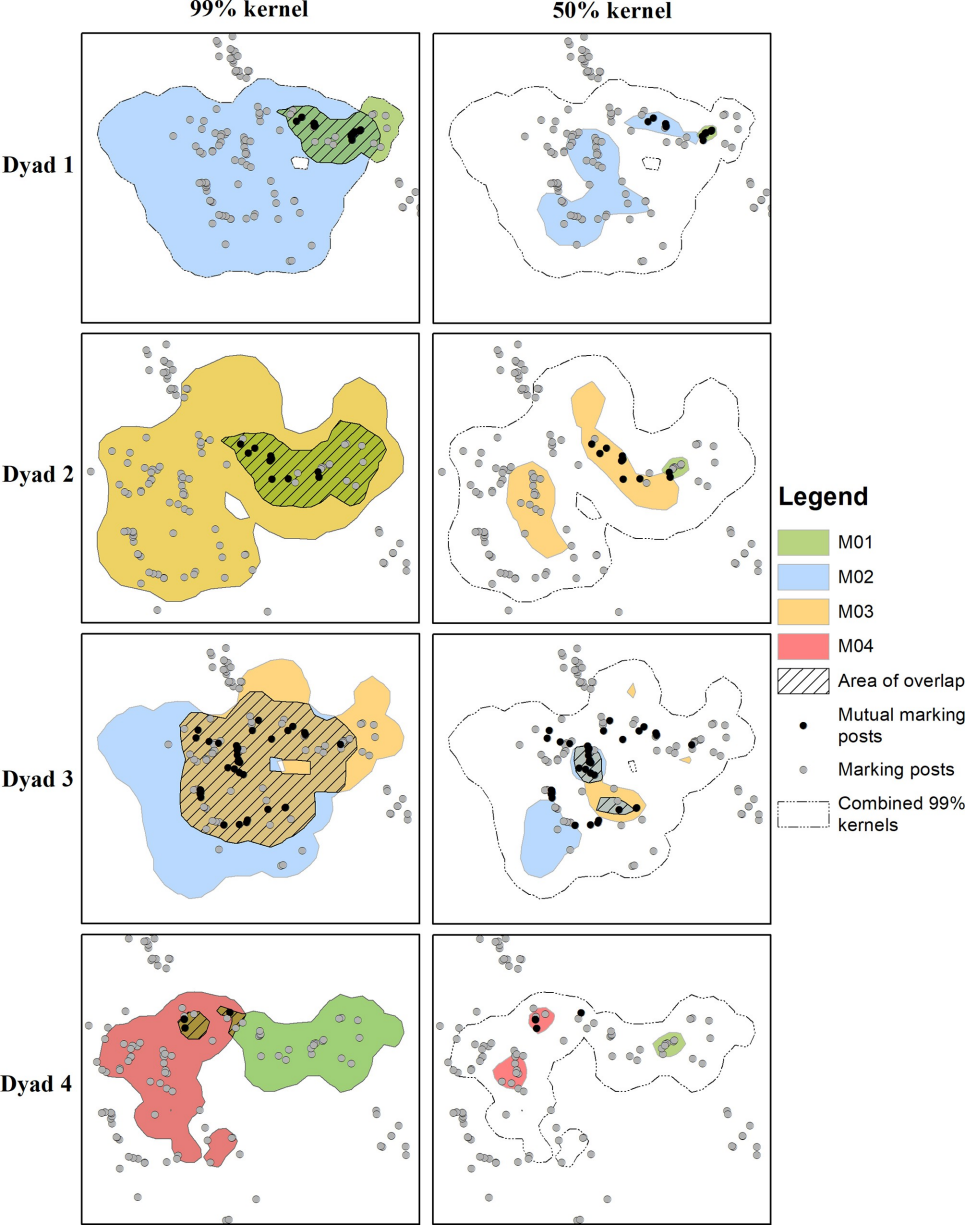
Space us and overlap of the 99% and 50% kernels for the individuals in each dyad and the location of known and mutual marking posts.

Across the four dyads the 99% kernels ranged from 253 km^2^ to 1,903 km^2^ and the 50% kernels ranged from 11 km^2^ to 359 km^2^ (Table 2). For all the dyads, the 99% kernels overlapped but the amount of overlap ranged from 10% to 100% where in Dyad 2 the 99% kernel of individual M01 fell completely within the 99% kernel of individual M03 (Fig 1). Only the core areas (50% kernel) of Dyad 3 had an extensive area of overlap (28% and 48%), whereas the cores of the other dyads did not overlap or the area of overlap was minimal (< 4%).

We found 125 marking posts in the study area and the number of mutual marking posts per dyad ranged from 4 to 37 with the average number of visits to these marking posts ranging from 1 to 46.16 (Table 3). For the three dyads that had the most extensive spatial overlap and the largest number of mutual marking posts (Dyads 1, 2 and 3) the average time that an individual was within 500m of a mutual marking post did not vary significantly between individuals in the same dyad, ranging from 5.06 ± 4.56 hours to 7.95 ± 5.85 hours (mean ± standard deviation; Table 3). The data were not normally distributed and the median for the same three dyads ranged from 2.52 ± 2.35 hours to 7.02 ± 6.01 hours (median ± median absolute deviation). For all the dyads, the average time between visits of a mutual marking post varied significantly between the individuals (Table 3). In the case of Dyad 1 and 2, individual M01, who we classified as territorial, visited mutual marking posts more frequently compared to individuals M02 and M03, neither of which were strictly territorial (Table 1). The time between different individuals visiting the same mutual marking post ranged from 3.11 ± 4.01 days to 13.64 ± 8.51 days and did not differ significantly across the dyads (Table 3).

**Table 3:** Summary of marking posts visits by male cheetahs in each dyad.

Dyad	Total no. marking posts visited in 99% kernel overlap With a proximity of 500m	ID	No. marking posts visited	No. mutual marking posts visited	Number of visits to mutual marking posts (mean)	Average time per individual at mutual marking posts (hours) ^˄^		Average time per individual between visits of mutual marking posts (days) ^˄^		Time between different individuals visiting mutual marking posts (days) ^˄^	
1	20	M01	20	12	46.16	7.95 ± 5.85 (7.02 ± 6.01)	W = 4384 P = 0.025	3.29 ± 4.65 (1.99 ± 1.82)	W = 836 P = <0.001	7.61 ± 8.78 (3.86 ± 4.24)	W = 50 P = 0.149
		M02	12		1.83	6.67 ± 8.66 (2.52 ± 2.35)		24.42 ± 22.54 (15.18 ± 20.68)		3.11 ± 4.01 (1.34 ± 1.18)	
2	24	M01	23	11	14.73	5.72 ± 4.78 (3.89 ± 4.37)	W = 1950 P = 0.369	8.65 ± 9.26 (6.34 ± 4.93)	W = 70 P = <0.001	13.04 ± 11.43 (9.58 ± 8.74)	W = 73 P = 0.402
		M03	12		2.45	5.06 ± 4.56 (2.74 ± 3.44)		41.20 ± 47.13 (24.98 ± 36.26)		8.06 ± 7.53 (6.11 ± 7.81)	
3	64	M02	43	37	5.35	7.59 ± 8.52 (4.51 ± 4.99)	W = 11085 P = 0.177	26.65 ± 47.78 (11.96 ± 15.96)	W = 768 P = <0.001	13.64 ± 8.51 (16.10 ± 11.64)	W = 189 P = 0.368
		M03	58		3.32	5.22 ± 4.38 (3.97 ± 4.12)		15.78 ± 21.94 (7.37 ± 9.14)		11.99 ± 10.16 (7.62 ± 7.91)	
4	7	M01	5	4	1	1.15 ± 0.54 (0.91 ± 0.90)	W = 141 P = <0.001	-	NA	25.89˜	NA
		M04	6		10.17	7.72 ± 6.73 (6.74 ± 5.45)		5.60 ± 5.14 (5.09 ± 2.74)		0.87˜	

^˄^Median ± median absolute deviation are provided in the parenthesis

˜Only 1 occasion

### Dynamic interaction

**Fig 2:**
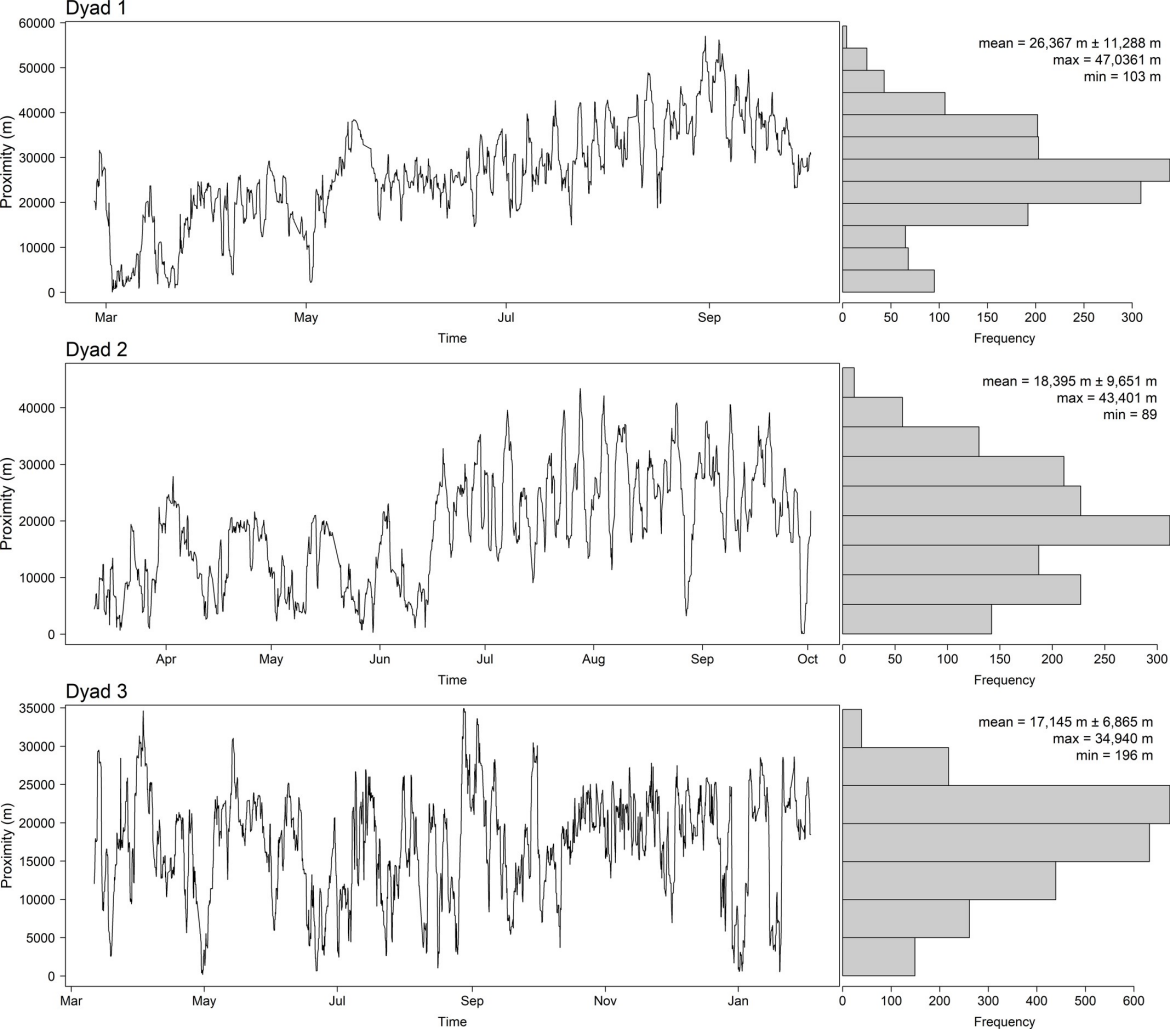
Proximity plot for the three male cheetah dyads that had possible encounters based on GPS collar data collected every 3 hours.

For three of the four dyads (Dyads 1, 2 and 3) the possibility of individuals within each dyad encountering each other was high as they overlapped extensively in space and had a large number of mutual marking posts. For these three dyads we explored their simultaneous movements and calculated the proximity between each individual within a dyad. The individuals within the three dyads did on occasions come into close proximity to one another as can be seen in the animation provided in the S1 Movie. The minimum distance between individuals in a dyad ranged from 89 m to 196 m but the average proximity between individuals ranged from 17,145 ± 6,865 m to 26,367 ± 11,288 m (Fig 2). Possible encounters were classified according to four different thresholds and we detected four possible encounters with a proximity threshold of <500m, 11 with a proximity threshold of <1000m, 21 with a proximity threshold of <1500m and 25 with a proximity threshold of <2000m (Table 4). Possible encounters were more likely to occur at night than during the day (χ = 8, df = 1, p = 0.005) and occurred most frequently at 21hr and midnight. Of the 25 possible encounters, 64% (n = 16) occurred within the core area of one individual, 28% (n = 7) occurred where the 50% kernels overlapped and two possible encounters in Dyad 3 did not occur in any of the core areas. Eleven (44%) of the possible encounters occurred within 500m of a known marking post.

**Table 4:** Summary of possible encounters between male cheetahs in the Maasai Mara, Kenya, based on GPS-collar data set to collect data every 3 hours.

Proximity threshold	Encounter	Dyad	Minimum proximity	Duration (number of simultaneous GPS fixes)	Time	Time of day	Core	Distance to nearest marking post (m)
< 500m	1	2	89	9	18:00	Day	M03	1,506
	2	1	103	10	21:00	Night	M01	218
	3	3	196	8	06:00	Night	-	391
	4	2	327	2	00:00	Night	M03	268
< 1000m	5	3	537	2	00:00	Night	Both	726
	6	3	603	7	12:00	Day	Both	607
	7	3	648	6	18:00	Day	Both	635
	8	3	662	6	09:00	Day	Both	182
	9	2	697	1	21:00	Night	M01	391
	10	2	755	3	21:00	Night	M03	2,791
	11	1	902	2	00:00	Night	M02	152
< 1500m	12	1	1009	7	00:00	Night	M02	1,456
	13	1	1017	3	03:00	Night	M01	308
	14	2	1024	4	06:00	Night	M01	582
	15	3	1047	2	03:00	Night	M03	1,418
	16	2	1121	1	00:00	Night	Both	305
	17	2	1213	1	21:00	Night	M03	590
	18	2	1233	1	21:00	Night	M01	674
	19	2	1233	1	06:00	Night	Both	531
	20	1	1238	6	09:00	Day	M01	30
	21	3	1371	2	06:00	Night	-	228
<2000m	22	2	1646	1	21:00	Night	M01	496
	23	3	1690	1	00:00	Night	Both	807
	24	2	1854	1	12:00	Day	M03	780
	25	1	1877	1	18:00	Day	M02	1,443

### Encounter outcomes

For possible encounters with proximity thresholds of <500m, <1000m and <1500m the distance between males was overall significantly less during the period 3 to 6 hours after a possible encounter, compared to the 3 and 6 hours before a possible encounter (Table 5). In other words, rather than moving away from each other, male cheetahs stayed in close proximity to each other for up to 6 hours after a possible encounter.

**Table 5:** Distance between individuals within a dyad before and after encounters with four different proximity thresholds. Bold indicates significant results.

Proximity threshold	Lag (hours)	Mean distance between individuals (m)		Paired t-test results		
		Before	After	T	df	p
<500m	3	2,480 ± 1,533	1,288 ± 1,151	4.523	3	**0.020**
	6	5,269 ± 1,213	1,834 ± 2,433	4.271	3	**0.023**
	12	9,244 ± 5,447	3,246 ± 2,571	1.976	3	0.143
	24	16,386 ± 6,220	5,308 ± 5,316	1.447	3	0.285
<1000m	3	2,433 ± 1,683	1,350 ± 842	2.203	10	0.052
	6	4,318 ± 2,430	2,227 ± 1,989	3.771	10	**0.004**
	12	5,837 ± 4,544	3,728 ± 2,368	1.477	10	0.171
	24	8,710 ± 6,555	7,236 ± 6,314	0.481	8	0.644
<1500m	3	2,849 ± 1,920	1,947 ± 1,091	2.242	20	**0.036**
	6	4,365 ± 2,756	2,825 ± 1,986	2.611	20	**0.017**
	12	5,839 ± 4,099	4,067 ± 2,195	1.816	20	0.084
	24	7,700 ± 6,025	7,123 ± 5,192	0.466	17	0.647
<2000m	3	3,066 ± 2,031	2,470 ± 1,812	1.558	24	0.132
	6	4,380 ± 2,650	3,532 ± 2,898	1.367	24	0.184
	12	5,615 ± 3,918	4,841 ± 2,892	0.818	24	0.422
	24	8,035 ± 6,242	7,406 ± 4,798	0.535	21	0.600

For the distance to the encounter location, distance travelled and tortuosity we wanted to determine whether there was individual variation within each dyad. However, because of the paucity in the number of possible encounters that were detected per dyad we were only able to carry out the analysis for possible encounters with a proximity threshold <2000m. In general, cheetahs were closer to the encounter location after a possible encounter compared to before for all four time lags, apart from individual M03 in Dyad 3 where the opposite trend was observed, however none of the results were significant (S1 Table). Similarly, cheetahs travelled less after a potential encounter compared to before, apart from individual M03 in Dyad 3 where the opposite trend was observed. Some of the results, especially at the 12hr and 24hr lag were significant for Dyad 1 and 2 (S1 Table).

**Fig 3:**
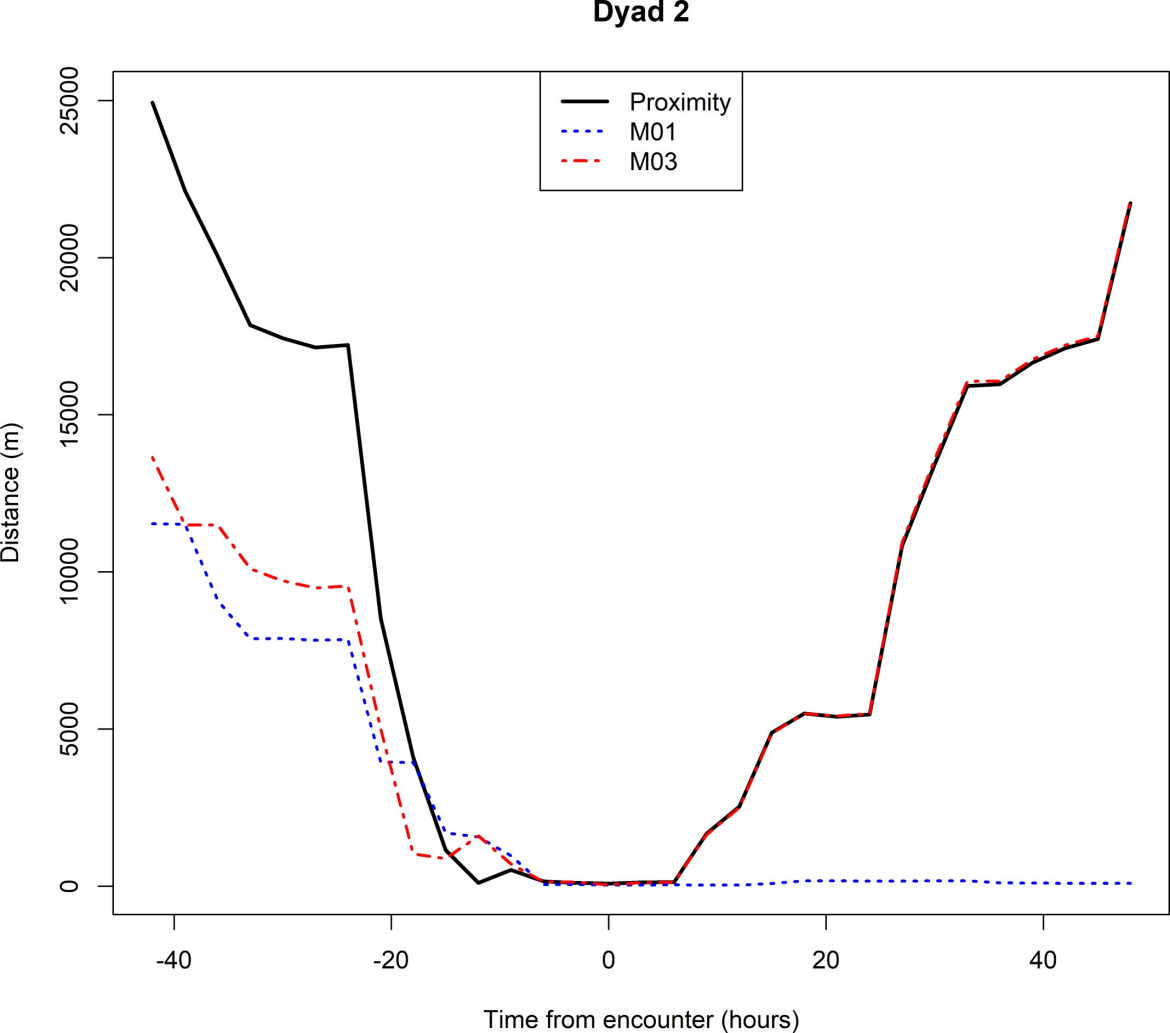
Plot of the proximity between M01 and M03 (Dyad 2) and their respective distances to the encounter locations that likely resulted in the death of M01. An animation of this interaction can be found in the S2 Movie.

On the 11^th^ February 2017 the collar on M04 stopped transmitting but when the team visited the last location sent by the collar, neither cheetah nor collar could be found and the individual has not been seen since. On the 1^st^ October 2017 the collar on M01 stopped transmitting data after the collar data showed that individuals M01 and M03 had come within 89m of each other. The team went to the last GPS coordinate that was transmitted by the collar and found the remains of M01 70m from the last GPS fix sent by the collar. Upon inspecting the carcass, a puncture wound was found on the left side of the skull. Based on the circumstantial evidence, we believe that the death of M01 was either a direct or an indirect result of an aggressive interaction between him and the 5-male coalition (M03). Interestingly, three months prior to this encounter M03 and his coalition mates started establishing a territory approximately 25km southwest from M01’s territory. Two days before the encounter the coalition travelled 25 km to the encounter location, spent 12 hours within 150 m of M01 and then travelled 19km straight back to the core of their territory. M03 did not return to the vicinity of the encounter between the 1^st^ October 2017, when the encounter took place, and 3^rd^ February 2018, when M03’s collar was removed. The closest they came to M01’s territory during that time was approximately 10 km (Fig 3 and the animation in S2 Movie).

## Discussion

Using GPS collar data we documented static and dynamic interactions between male cheetahs in Kenya’s Maasai Mara and investigated the outcomes of these interactions in terms of movement behaviour and mortalities. As we predicted, male cheetahs showed extensive spatial overlap of the 99% kernels. This high degree of overlap observed in the Maasai Mara could be related to the pattern of prey availability [43], although we do not have the data to test this. However, apart from one dyad, there was little overlap of core areas (50% kernels) and it could be that core areas are defended more intensively than the peripheral areas [29]. Similar to observations in other areas, marking posts were frequently visited by males [29, 44] and this could indicate the mechanism that results, despite the extensive spatial overlap, in the rarity of occasions when members of a dyad were in close proximity [27]. Interestingly, our results show that possible encounters were most likely to take place in the core area of one individual of a dyad or where cores of both individuals overlapped. We also found that, similar to African wild dogs (*Lycaon pictus*), possible encounters occurred more at night than during the day [17]. While cheetahs, like African wild dogs, are predominantly diurnal they can be active at night [45] and nocturnal activity for males has been found to be considerably higher than for females [46]. Data from camera traps set at marking posts found that visits occurred more at night than during the day ([44]; KK unpublished data) suggesting that male nocturnal activity is partly driven by patrolling behaviour which is probably why encounters predominantly took place at night.

In some species, including African wild dogs and white-faced capuchins (*Cebus capucinus*), avoidance behaviours, characterised by an increase in distance and speed travelled post-encounter, were observed as a result of interactions between different groups [17, 18]. However our results, in contrast to our predictions, did not show avoidance behaviour post-encounter as males stayed in close proximity to each other 3–6 hours after a potential encounter. It is possible that males stayed in close proximity to each other, as part of a territorial defense strategy, if a recent scent of a conspecific was detected. This behaviour has been observed in dwarf mongoose (*Helogale parvula*) groups, who moved slower and covered shorter distances in the hour following the encounter of rival faeces at a latrine site within their territory [16] and red fox (*Vulpes vulpes*) males who spent more time in scent-marked areas [47]. Alternatively, males could come into close proximity to one another if they are attracted to a resource, such as a female in oestrus [27, 48]. Cheetahs exhibit a high rate of multiple paternity [49] so it is possible that multiple males stay in the vicinity of a receptive female with the hope of getting a chance to mate. These encounters could however result in fatalities if the removal of competition increases future mating opportunities [50]. If encounters occur as a result of access to a receptive female rather than to a static, long-term resource such as a territory then this could explain why the five-male coalition did not take-over the territory of individual M01 after he died.

Aggressive interactions with fatal consequences are not uncommon in cheetahs. Caro [22] reported three cases in Serengeti where singletons were killed by coalitions (all three-male coalitions). Similarly, Mills and Mills [27] found that 50% of male-male encounters recorded in the Kgalagadi Transfrontier Park in Botswana/South Africa resulted in death. To our knowledge, fatal interactions have not been observed between female cheetahs. This could explain why male mortality is higher and life expectancy lower for males compared to females [51, 52] resulting in a female biased sex ratio [52]. For some species, such as voles (*Microtus oeconomus*), lions and grizzly bear (*Ursus arctos*), the removal of males, through either displacement or mortality, has a negative effect on population growth as a result of increased infanticide [53–55]. Infanticide has however not been observed amongst cheetahs [56] possibly because it rarely occurs in predominantly solitary species [57]. The removal of males could however have other population-level consequences [58] but the impact of male mortality on population dynamics in cheetahs is unclear.

Static and dynamic interactions can play a role in disease transmission [3, 4]. In the Mara-Serengeti ecosystem there is a relatively high prevalence of mange [59, 60] and in Southern Africa cheetahs have been positively tested for feline coronavirus (FCoV) and feline panleukopenia virus (FPV), which can be highly contagious and fatal [61, 62]. Pathogens such as these can easily spread through faeces and other bodily fluids, which are deposited and investigated by male cheetahs at marking posts. This could explain why in 2015 several males in the Maasai Mara, who overlapped spatially, died of a yet unknown disease within a short space of time [63]. We suggest that future epidemiological research should investigate the role of scent marking posts and movement in disease transmission [64].

Here we give a descriptive analysis of the static and dynamic interactions between male cheetahs and the outcomes of these encounters. Despite the clear patterns that were observed, there are several caveats that warrant discussion. Firstly, we were only able to use data from four collared males, one of which was part of a 5-male coalition. It is therefore possible that other uncollared individuals, including the other members of the 5-male coalition, could have influenced the results. Secondly, because of the resolution of the collar data we might have missed visits to marking posts and we inferred when interactions took place rather than being able to detect actual interaction (apart from one occasion). Our results are therefore likely to be on the conservative side and we suggest that future studies use higher resolution data and/or proximity loggers to investigate actual interactions between individuals (e.g. [17, 65]). However, even with a relatively coarse resolution of data and only a small number of individuals we managed to investigate interactions and subsequent outcomes between males giving a first detailed insight into intraspecific interactions in cheetah.

## Supporting information

S1 Movie Animation of the simultaneous movement of individuals M01, M02, M03 and M04. (MOV)

S2 Movie Animation of the simultaneous movement of individuals M01 and M03 which resulted in the mortality of M01. (MOV)

S1 Table Summaries for each dyad of the distance to the encounter location, distance travelled and tortuosity before and after a possible encounters with proximity threshold of <2000m. (PDF)
