# Supplementary material for: Using GPS collars to investigate the frequency and behavioural outcomes of intraspecific interactions among carnivores: A case study of male cheetahs in the Maasai Mara, Kenya
Source: PLoS One. 2019 Apr 3;14(4):e0213910. doi: 10.1371/journal.pone.0213910 (PMC6447186; doi:10.1371/journal.pone.0213910)
Supplement: S1 Table — (PDF) [file pone.0213910.s003.pdf]

Using GPS collars to investigate the frequency and behavioural outcomes of intraspecific interactions among carnivores: A case study of male cheetahs in the Maasai Mara, Kenya

Femke Broekhuis[1,2*], Emily K. Madsen[3], Kosiom Keiwua[1] and David W. Macdonald[2]

[1] Kenya Wildlife Trust, P.O. Box 86-00502 Karen, Nairobi, Kenya

[2] Wildlife Conservation Research Unit, Department of Zoology, University of Oxford, Recanati-Kaplan Centre, Tubney, OX13 5QL, UK

[3] Centre for Biodiversity and Environmental Research, University College London, Gower Street, London, WC1E 6BT, United Kingdom

*Corresponding author: Femke Broekhuis

Email: femke.broekhuis@gmail.com

Phone: +254 701 158 737

Journal: PLOS ONE

Table S1 Summary for Dyad 1 of the distance to the encounter location, distance travelled and tortuosity before and after a possible encounters with proximity threshold of <2000m.

**ID**	**Metric**	**Lag (hours)**	**Before**	**After**	**Results**
M01	Distance to encounter location (m)	3	2,620 ± 2,184	913 ± 355	V = 16, P = 0.31
		6	4,348 ± 3,988	2,221 ± 2,185	V = 13, P = 0.69
		12	4,292 ± 4,009	2,718 ± 3,389	V = 16, P = 0.31
		24	6,028 ± 3,568	4,471 ± 3,948	V = 18, P = 0.16
	Distance travelled (m)	3	2,534 ± 2,052	1,436 ± 2,017	t_5 = 1.02, P = 0.35
		6	4,222 ± 3,665	3,226 ± 3,381	t_5 = 0.60, P = 0.57
		12	5,089 ± 3,320	3,965 ± 3,959	t_5 = 0.72, P = 0.51
		24	11,176 ± 6,867	5,256 ± 3,211	t_5 = 2.97, P = **0.03**
	Tortuosity	6	0.99 ± 0.01	0.87 ± 0.30	t_5 = 1.00, P = 0.36
		12	0.84 ± 0.29	0.86 ± 0.27	t_5 = -0.08, P = 0.94
		24	0.60 ± 0.26	0.43 ± 0.37	t_3 = 0.34, P = 0.75
M02	Distance to encounter location	3	1,342 ± 828	920 ± 446	V = 16, P = 0.31
		6	2,384 ± 1,751	1,146 ± 836	V = 17, P = 0.22
		12	2,754 ± 1,778	1,181 ± 637	V = 19, P = 0.09
		24	5,405 ± 4,311	2,082 ± 1,372	V = 16, P = 0.31
	Distance travelled	3	1,215 ± 872	778 ± 931	t_5 = 1.75, P = 0.14
		6	2,488 ± 1,901	799 ± 916	t_5 = 2.23, P = 0.08
		12	3,058 ± 1,628	1,204 ± 929	t_5 = 2.72, P = **0.04**
		24	7,398 ± 3,383	3,238 ± 2,924	t_5 = 2.09, P = 0.09
	Tortuosity	6	0.94 ± 0.89	0.27 ± 0.89	t_5 = 0.57, P = 0.59
		12	0.89 ± 0.13	0.81 ± 0.23	t_5 = 0.55, P = 0.60
		24	0.68 ± 0.36	0.67 ± 0.37	t_5 = 0.03, P = 0.98

Table S2 Summary for Dyad 2 of the distance to the encounter location, distance travelled and tortuosity before and after a possible encounters with proximity threshold of <2000m.

ID	Metric	Lag (hours)	Before	After	Results
M01	Distance to encounter location (m)	3	2,096 ± 1,617	1,731 ± 1,676	V = 42, P = 0.46
		6	2,703 ± 1,919	2,722 ± 3,033	V = 40, P = 0.58
		12	3,827 ± 2,699	3,159 ± 2,749	V = 41, P = 0.52
		24	4,776 ± 3,395	3,293 ± 2,621	V = 45, P = 0.32
	Distance travelled (m)	3	1,951 ± 1,657	1,465 ± 1,567	t_{10} = 1.62, P = 0.13
		6	3,427 ± 2,812	2,563 ± 3,028	t_{10} = 1.25, P = 0.23
		12	5,565 ± 3,453	3,792 ± 3,963	t_{10} = 1.33, P = 0.21
		24	9,183 ± 3,940	4,736 ± 4,265	t_{10} = 4.01, P < **0.00**
	Tortuosity	6	0.89 ± 0.14	0.97 ± 0.05	t_{10} = -1,74, P = 0.11
		12	0.76 ± 0.24	0.75 ± 0.27	t_{10} = -0.09, P = 0.92
		24	0.50 ± 0.29	0.72 ± 0.42	t_{10} = -1.45, P = 0.18
M03	Distance to encounter location	3	1,586 ± 1,533	1,327 ± 999	V = 40, P = 0.58
		6	2,017 ± 1,604	1,432 ± 1,034	V = 44, P = 0.37
		12	2,867 ± 1,028	2,718 ± 1,172	V = 35, P = 0.89
		24	3,455 ± 6,327	2,304 ± 2,864	V = 13, P = 0.08
	Distance travelled	3	1,360 ± 1,687	1,011 ± 947	t_{10} = 0.76, P = 0.46
		6	2,002 ± 1,705	1,201 ± 1,036	t_{10} = 1.51, P = 0.16
		12	4,237 ± 2,543	2,638 ± 953	t_{10} = 2.07, P = 0.06
		24	6,007 ± 2,817	5,696 ± 4,117	t_{10} = 0.23, P = 0.82
	Tortuosity	6	0.92 ± 0.11	0.87 ± 0.22	t_{10} = -1,74, P = 0.11
		12	0.84 ± 0.11	0.92 ± 0.09	t_{10} = -0.09, P = 0.92
		24	0.61 ± 0.23	0.94 ± 0.33	T_5 = -1.81, P = 0.12

Table S3 Summary for Dyad 3 of the distance to the encounter location, distance travelled and tortuosity before and after a possible encounters with proximity threshold of <2000m.

ID	Metric	Lag (hours)	Before	After	Results
M02	Distance to encounter location (m)	3	1,927 ± 2,268	718 ± 712	V = 24, P = 0.46
		6	3,104 ± 3,959	1,126 ± 1.009	V = 24, P = 0.46
		12	3,372 ± 4,067	1,634 ± 1,512	V = 24, P = 0.46
		24	6,332 ± 5,945	4,773 ± 5,121	V = 22, P = 0.64
	Distance travelled (m)	3	1,791 ± 2,151	540 ± 672	t_7 = 1.58, P = 0.16
		6	3,403 ± 3,804	1,025 ± 1,090	t_7 = 1.79, P = 0.11
		12	3,954 ± 3,899	1,731 ± 1,696	t_7 = 1.44, P = 0.19
		24	8,061 ± 6,251	3,245 ± 3,122	t_7 = 2.21, P = 0.06
	Tortuosity	6	0.88 ± 0.16	0.82 ± 0.25	t_7 = 0.55 , P = 0.59
		12	0.85 ± 0.23	0.74 ± 0.30	t_7 = 1,13, P = 0.29
		24	0.83 ± 0.14	0.58 ± 0.38	t_7 = 1.63, P = 0.20
M03	Distance to encounter location	3	779 ± 392	915 ± 550	V = 10, P = 0.31
		6	1,096 ± 461	1,187 ± 1,062	V = 16, P = 0.84
		12	1,664 ± 1,161	2,830 ± 1,228	V = 5, P = 0.08
		24	3,333 ± 2,190	3,095 ± 1,426	V = 21, P = 0.74
	Distance travelled	3	698 ± 804	760 ± 704	t_7 = 0.55, P = 0.59
		6	1,599 ± 1,533	1,201 ± 1,358	t_7 = -0.25, P = 0.81
		12	2,828 ± 2,256	3,638 ± 1,944	t_7 = -0.81, P = 0.44
		24	5,373 ± 2,705	4,964 ± 2,487	t_7 = 0.41, P = 0.68
	Tortuosity	6	0.84 ± 0.34	0.87 ± 0.15	t_7 = -0.27 , P = 0.79
		12	0.91 ± 0.09	0.78 ± 0.16	t_7 = 1.79, P = 0.11
		24	0.63 ± 0.31	0.51 ± 0.17	t_7 = -0.24, P = 0.82
